# ID 112 - Evidências Clínicas da Beta-Agalsidase Versus Alfagalsidase no Tratamento da Doença de Fabry Clássica: um cenário de incorporação no SUS

**DOI:** 10.5327/2237-9622.2025.v34s1.112

**Published:** 2025-11-25

**Authors:** Ariane Gonçalves Silva de Araujo, Fernanda Stumpf Tonin, Astrid Wiens Souza

**Keywords:** doença de Fabry, Avaliação de Tecnologias em Saúde, terapia de reposição de enzimas

## Abstract

**Introdução:**

A doença de Fabry clássica é um distúrbio metabólico ultrarraro com importante impacto na qualidade de vida, podendo reduzir a expectativa de vida em até 20 anos em pacientes não tratados. E apesar das terapias de reposição enzimática (TRE) como beta-agalsidase e alfagalsidase ter potencial para reduzir os danos, o acesso a elas é considerado uma barreira no manejo eficaz desses pacientes. Este estudo realizou uma síntese de evidências clínicas para subsidiar a decisão sobre a incorporação da beta-agalsidase como alternativa terapêutica à alfagalsidase no SUS.

**Método:**

Foi conduzida uma revisão sistemática nas bases PubMed, Embase, Cochrane CENTRAL e LILACS, complementada por busca manual. Buscou-se identificar revisões sistemáticas, ensaios clínicos randomizados e estudos observacionais comparativos, com pacientes com doença de Fabry de fenótipo clássico, incluindo aqueles com fenótipo misto ou não informado, além de estudos de troca terapêutica (switching) entre as TREs. A avaliação do risco de viés dos estudos incluídos foi realizada pela ferramenta Risk of Bias (RoB 2) nos ensaios clínicos e pela Risk Of Bias In Non-randomized Studies of Interventions (ROBINS-I) nos estudos observacionais. A qualidade metodológica das revisões sistemáticas foi avaliada pela ferramenta (AMSTAR-2), e a qualidade das evidências pelo (GRADE).

**Resultados:**

No total, 22 estudos foram incluídos. Cinco estudos compararam diretamente beta-agalsidase e alfagalsidase (um ensaio clínico randomizado e quatro estudos observacionais). Dezessete estudos analisaram desfechos de troca terapêutica (uma revisão sistemática e 16 estudos observacionais). Nenhum estudo avaliou desfechos de sobrevida. Desfechos de qualidade de vida, mortalidade, eventos cerebrovasculares e adversos foram reportados apenas em estudos de troca terapêutica. Não houve mudanças na qualidade de vida após a troca entre TREs, e 13 mortes foram relatadas em um acompanhamento de longo prazo. Eventos adversos foram pouco reportados e, em estudos mais recentes, não foram identificados eventos adversos sérios. Um único estudo de switch relatou redução do risco de acidente vascular cerebral e ataque isquêmico transitório na troca entre TREs (RR 0,20; IC 95% 0,03-0,29). Eventos clínicos foram reportados tanto por comparação direta quanto por switching, sendo similares entre beta-agalsidase e alfagalsidase (HR=0,96; IC 95% 0,59-1,57; p=0,87; qualidade da evidência baixa). Desfechos relacionados à função renal (p=0,708) e cardiovascular (p=0,150) foram semelhantes entre as TREs, embora a intensidade dessas reduções variou nos estudos (qualidade da evidência muito baixa). O desenvolvimento de anticorpos contra TRE foi observado apenas em homens com doença de Fabry, sendo mais frequente com beta-agalsidase (OR=2,8; IC 95% 1,02-7,88; p=0,041; qualidade da evidência baixa).

**Conclusão:**

Não foram encontradas diferenças significativas entre beta-agalsidase e alfagalsidase em termos de eficácia, segurança ou impacto na qualidade de vida. A formação de anticorpos contra beta-agalsidase, embora mais frequente, ainda não tem significância clínica estabelecida. As variações nos resultados parecem estar associadas à heterogeneidade entre os pacientes. Estudos adicionais, especialmente de não inferioridade ou equivalência, são necessários para confirmar esses achados.

